# Longitudinal Bedside Assessments of Brain Networks in Disorders of Consciousness: Case Reports From the Field

**DOI:** 10.3389/fneur.2018.00676

**Published:** 2018-08-21

**Authors:** Corinne A. Bareham, Judith Allanson, Neil Roberts, Peter J. A. Hutchinson, John D. Pickard, David K. Menon, Srivas Chennu

**Affiliations:** ^1^Department of Clinical Neurosciences, University of Cambridge, Cambridge, United Kingdom; ^2^Cambridge University Hospitals NHS Foundation Trust, Cambridge, United Kingdom; ^3^Sawbridgeworth Medical Services, Jacobs & Gardens Neuro Centres, Sawbridgeworth, United Kingdom; ^4^Division of Anaesthesia, University of Cambridge, Cambridge, United Kingdom; ^5^School of Computing, University of Kent, Canterbury, United Kingdom

**Keywords:** consciousness, electroencephalography, brain networks, longitudinal assessment, disorders of consciousness, minimally conscious state, unresponsive wakefulness state

## Abstract

Clinicians are regularly faced with the difficult challenge of diagnosing consciousness after severe brain injury. As such, as many as 40% of minimally conscious patients who demonstrate fluctuations in arousal and awareness are known to be misdiagnosed as unresponsive/vegetative based on clinical consensus. Further, a significant minority of patients show evidence of hidden awareness not evident in their behavior. Despite this, clinical assessments of behavior are commonly used as bedside indicators of consciousness. Recent advances in functional high-density electroencephalography (hdEEG) have indicated that specific patterns of resting brain connectivity measured at the bedside are strongly correlated with the re-emergence of consciousness after brain injury. We report case studies of four patients with traumatic brain injury who underwent regular assessments of hdEEG connectivity and Coma Recovery Scale-Revised (CRS-R) at the bedside, as part of an ongoing longitudinal study. The first, a patient in an unresponsive wakefulness state (UWS), progressed to a minimally-conscious state several years after injury. HdEEG measures of alpha network centrality in this patient tracked this behavioral improvement. The second patient, contrasted with patient 1, presented with a persistent UWS diagnosis that paralleled with stability on the same alpha network centrality measure. Patient 3, diagnosed as minimally conscious minus (MCS–), demonstrated a significant late increase in behavioral awareness to minimally conscious plus (MCS+). This patient's hdEEG connectivity across the previous 18 months showed a trajectory consistent with this increase alongside a decrease in delta power. Patient 4 contrasted with patient 3, with a persistent MCS- diagnosis that was similarly tracked by consistently high delta power over time. Across these contrasting cases, hdEEG connectivity captures both stability and recovery of behavioral trajectories both within and between patients. Our preliminary findings highlight the feasibility of bedside hdEEG assessments in the rehabilitation context and suggest that they can complement clinical evaluation with portable, accurate and timely generation of brain-based patient profiles. Further, such hdEEG assessments could be used to estimate the potential utility of complementary neuroimaging assessments, and to evaluate the efficacy of interventions.

## Introduction

Recent years have seen substantial advances in the research and development of both behavioral tools and imaging methods to detect the level of awareness in patients with prolonged disorders of consciousness (pDOC), defined as those persisting 4 weeks or more after injury (1). Nonetheless, making an accurate clinical diagnosis remains challenging with the most recent figures indicating a misdiagnosis rate of almost 40%, when based on clinical consensus (2).

One of the factors contributing to this rate of misdiagnosis is the lack of a standardized diagnostic tool, or a gold standard for establishing the state of consciousness of a patient. The Coma Recovery Scale-Revised (CRS-R) (3) is considered the most valid scale for the systematic assessment of behavioral awareness in these patients (4) and has helped to identify those patients that have been misdiagnosed based on clinical examination (1). The CRS-R has subscales of assessment along different dimensions of behavioral responsiveness, which aim to distinguish those patients that show reflexive responses only (Unresponsive Wakefulness State/Vegetative State; UWS/VS), to those who show a degree of awareness with/without command following (Minimally Conscious State; MCS-/MCS+ respectively), to those who have emerged from minimal consciousness, as evidenced by functional object use and/or functional and accurate communication (Emerged from Minimally Conscious State; EMCS). Unfortunately, the 40% misdiagnosis rate comes from a large proportion of the MCS patients misdiagnosed as UWS in the absence of systematic behavioral assessment with methods like the CRS-R (2, 5).

Cases of some misdiagnosed patients have been found to demonstrate covert awareness using imaging techniques such as functional magnetic resonance imaging [fMRI (6, 7)] and electroencephalography [EEG (8)]. This highlights the potential utility of imaging techniques to assist with diagnosis in pDOC, especially considering that patients have typically sustained extremely severe brain injury that can lead to deficits to language, motor or general attention and arousal functioning that could lead to a failure to detect consciousness using a behavioral scale (9). Advances in neuroimaging, particularly fMRI, have found specific paradigms to measure cognition and have identified neural correlates, including prominently the Default Mode Network, which are associated with consciousness state in pDOC (10–13). Prominently, fMRI has been used to detect covert awareness and conscious experience in a significant minority of patients (6, 14).

The potential application of fMRI to develop a clinical diagnostic tool is problematic though, as it is not always readily available, feasible, or affordable, making it unsuitable for widespread application. While MRI assessments could be employed where suitable and feasible to build a detailed picture of brain structure and function, its use for regular patient follow-up is unlikely to be viable in the typical clinical context. Once patients leave the acute clinical care setting, they are often relocated to a rehabilitation center or nursing home for long-term care and rehabilitation. Typically, they are not followed up with regular fMRI assessments. Without regular follow-up of patients who might present variable and delayed improvements in behavior, it is difficult to determine the prognostic value of fMRI-based measures.

One promising avenue of neuroimaging research is the use of high-density EEG (hdEEG). Research has indicated that functional networks in the brain at rest, captured using various measures, are associated with the state of consciousness in pDOC (15–18). In particular, topologically structured networks of spectral connectivity in the alpha band have been shown to reflect consciousness levels in both patients (16, 18) and in healthy participants as they lose and regain consciousness during sedation (19). This research has repeatedly demonstrated that resting frontoparietal network connectivity might be an important EEG-based indicator of the state of consciousness. Most recently, Chennu et al. (18) showed that such network metrics estimated from resting state hdEEG could predict CRS-R diagnosis, 12-month outcomes and the presence/absence of frontoparietal metabolism [as measured by Positron Emission Tomography (20, 21)] in a large group of pDOC patients. Moreover, MCS patients who were misdiagnosed as UWS showed no differences in any of the measured hdEEG network metrics to the patients correctly diagnosed as MCS. This suggests that assessment of hdEEG networks could have both diagnostic and prognostic clinical value. One major benefit is that hdEEG assessments can be administered at the bedside, allowing for regular and repeated assessment to track the patient's trajectory of recovery. However, despite this potential, there is a substantial translational gap to viable clinical applications. The current national clinical guidelines for pDOC in the United Kingdom (UK) state that resting EEG cannot discriminate between UWS and MCS patients (1) and is not currently used routinely in a clinical setting for diagnostic purposes. This is primarily because, as it stands, there is no EEG-based clinical tool that has been developed, standardized or trialed in a large cohort of pDOC patients.

A related hurdle to the establishment of clinical utility is the fact that the vast majority of neuroimaging research in pDOC to date has taken a cross-sectional approach to compare patient diagnostic groups, using convenience sampling conducted at a particular point in time. This has generated valuable scientific insights about the nature of neural dysfunction in these states. However, inconsistencies between patients in regard to assessment methods can lead to poor validity or replicability, particularly if data collected is combined from multiple sites. Moreover, information from more fine-grained measures at the individual patient level can get lost using a cross-sectional approach. If the aim is to translate neuroimaging assessments from the bench to the clinic, we need to demonstrate that longitudinal monitoring in individual patients can produce consistent estimates of brain activity at the bedside. To address this translational gap, here we describe our prospective BETADOC (BEdside Test of Awareness for Disorders Of Consciousness) research study, which is amongst the first to apply a consistent method to collect hdEEG assessments and CRS-R assessments longitudinally in a group of pDOC patients, by systematically assessing them every 3 months over a period of 2 years. By conducting repeated and standardized brain network analyses of the data using a previously published pipeline (18), we track how fine-grained measures of resting state brain networks vary and progress alongside the behavioral trajectory of individual patients. This approach is enabling us to conduct longitudinal validation of hdEEG network metrics that we have previously shown to be associated with diagnosis and prognosis of consciousness in a cross-sectional study (18).

The overarching aim of the BETADOC study is to validate EEG-based metrics that accurately describe changes in the structure of hdEEG networks as individual patients recover over time. Using a longitudinal design, we can assess both the diagnostic and prognostic utility of hdEEG network metrics with multiple data points collected from each patient. In this original research report, we show preliminary results from four traumatic brain injury (TBI) patients in pDOC from the BETADOC project. The first patient progressed from UWS to MCS-, in contrast with the second patient who remained in UWS. The third patient transitioned from MCS– to MCS+, while the fourth patient remained in MCS–. We juxtapose the trajectories of individual patient's CRS-R scores with hdEEG visualizations and metrics identified a priori, based on prior research in an independent sample of patients (18). By demonstrating the robust relationship between these brain network metrics and CRS-R scores as patients progress through their individual trajectories, we provide a first sample of the evidence base required for viable clinical applications of resting state hdEEG assessments in pDOC.

## Materials and methods

### Ethics

This study was carried out in accordance with the recommendations of the UK National Health Service Research Ethics Committee for Cambridgeshire. The study protocol was approved by the committee (reference: 16/EE/0006). Patients' next-of-kin gave written informed consent prior to enrolment in the study, in accordance with the UK Mental Capacity Act 2005 and Declaration of Helsinki.

### Participants

We included two pDOC patients whose CRS-R scores reflected a transition to a progressively higher consciousness state across assessments, one from UWS to MCS- and one from MCS– to MCS+. These two patients were contrasted with two other patients whose CRS-R scores remained unchanged and reflected a stable UWS and MCS– state. All four patients had an etiology of traumatic brain injury following a road traffic accident (584–3,251 days since injury). As described further below, they were assessed using the CRS-R to ascertain behavioral diagnosis, and with high-density resting EEG to examine their brain activity. The same researcher (CAB) assessed each patient at their bedside every 3 months in the neurological center where they resided.

### Coma recovery scale-revised

The Coma Recovery Scale - Revised (CRS-R) is a behavioral assessment of awareness for pDOC (3). The 23-item scale is split into subscales that measure the auditory, visual, motor, oromotor/verbal, communication, and arousal levels of the patient. Some items are considered to be signs of consciousness, with the most complex items indicating EMCS. Formal comparison of available behavioral scales to assess awareness in this patient group indicated the CRS-R as one of the most reliable (22). The CRS-R was administered by the same trained neuropsychologist (CAB) with each patient at each time point. Time of day, and patient postural position was also noted although it was requested that patients were sitting upright in chair if possible.

### High-density EEG resting state

Fifteen minutes of resting state data was collected using a 128-channel saline electrode net [Electrical Geodesics (EGI)]. Data was collected at a sampling rate of 500 Hz and was later down-sampled to 250 Hz offline. Prior to EEG collection, the CRS-R was administered to assist with ensuring patients were awake with their eyes open. Patients' behaviors and EEG data were monitored online to ensure recordings were free from seizure activity.

**Figure 1:**
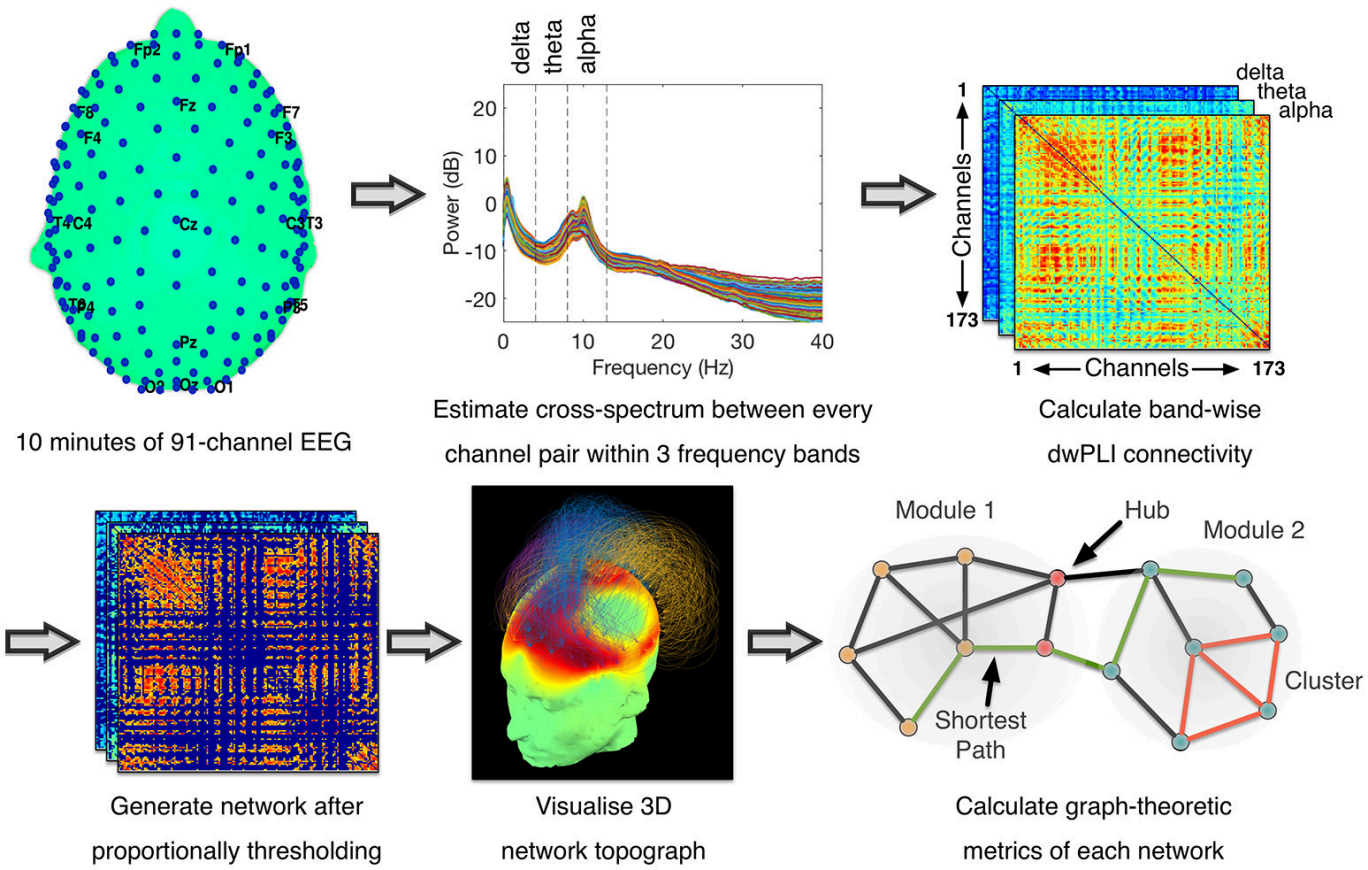
Data Processing Pipeline for Connectivity Analysis - Methodology was identical to (18). Cross-spectral density between pairs of channels was estimated using dwPLI. Resulting connectivity matrices were proportionally thresholded. Thresholded connectivity matrices were visualized as topographs, which combined information about the topography of connectivity with the modular topology of the network (see Figure 2 legend for details). Graph-theoretic metrics were then calculated after binarising the thresholded connectivity matrices.

The pre-processing and artifact rejection method was identical to that in Chennu et al. (18), as visualized in Figure 1. Briefly, data from electrodes near the eyes, face and neck was removed, leaving 91 electrodes for further analysis. Data was filtered at 0.5–45 Hz and then epoched to 10-second epochs. Each epoch thus generated was baseline-corrected relative to the mean voltage over the entire epoch. Data containing excessive eye movement or muscular artifact was rejected by a quasi-automated procedure: abnormally noisy channels and epochs were identified by calculating their normalized variance and then manually rejected or retained by visual inspection. After artifact rejection, there was on average 10.74 min of data (*SD* = 2.50) from each assessment for estimation of power and connectivity. This involved rejection of an average of 17% of the data in each assessment (*SD* = 19%). Independent Components Analysis (ICA) based on the Infomax ICA algorithm (23) was used to visually identify and reject noisy components (Mean = 41%, *SD* = 17%). Finally, previously rejected channels (Mean = 18%, *SD* = 10%) were interpolated using spherical spline interpolation, and data were re-referenced to the average of all channels.

Using a multitaper method with five Slepian tapers (24), spectral and cross-spectral decompositions within the canonical delta (0.5–4 Hz), theta (4–8 Hz) and alpha (8–13 Hz) frequency bands were computed at bins of 0.1 Hz. Spectral power values were normalized by dividing the power at each bin by the total power over all three bands and multiplying by 100 (17). Alongside, the cross-spectral decomposition was used to estimate the debiased weighted phase lag index (dwPLI) (25) metric of connectivity between every pair of electrodes. dwPLI minimizes the effects of volume conduction on the estimation of brain connectivity, and is further minimally biased at small sample sizes (25). Within each frequency band, dwPLI values at the peak frequency of the oscillatory signal across all channels were used to represent the connectivity between channel pairs. From each subject's dataset, the dwPLI values across all channel pairs were used to construct symmetric 91 × 91 dwPLI connectivity matrices for the delta, theta and alpha bands.

The dwPLI matrices thus constructed were thresholded proportionally to preserve 90–10% of the largest dwPLI values in steps of 2.5%. Specifically, at the 90% threshold, only the 10% of the weakest network edges were discarded. At the 10% threshold, 90% of the weakest edges were discarded. This lowest threshold of 10% ensured that the average degree was not smaller than 2 log(N), where N is the number of nodes in the network (i.e., *N* = 91). This in turn guaranteed that the resulting networks could be estimated (26). Further, graph connection densities within this range of thresholds have been shown to be sensitive to the estimation of “true” topological structure therein (27, 28).

After applying each of these thresholds, matrices were binarised, i.e., non-zero values were set to 1. These matrices were then modeled as networks with channels as nodes and binarised dwPLI values as connections between them. These networks were analyzed using graph theory algorithms to calculate a pre-defined set of summary metrics previously evaluated in an independent dataset (18)–clustering coefficient, characteristic path length, modularity, participation coefficient and modular span–at each value of the proportional threshold. The clustering coefficient of a network captures its local efficiency (26), while the characteristic path length measures the average topological distance between pairs of nodes in a graph, providing a measure of global efficiency (26). Modularity, calculated here using the Louvain algorithm (29), is a network metric that captures the degree to which the nodes of a network can be parcellated into densely connected, topologically distinct modules (30). Given a modular decomposition, the participation coefficient of a node is an inter-modular measure of its centrality (31). A larger standard deviation in participation coefficient of network nodes indicates a diversity of connectivity, and hence the presence of hub nodes that link many modules together in an efficient network. Here, we used the standard deviation of participation coefficients to measure network centrality as the presence of diversely connected nodes with central hubs (32, 33). Finally, modular span is average weighted topographical distance (over the scalp) spanned by a module identified in a network (16). Network metrics were averaged over all connection densities considered, to reduce them down to scalar values when plotting them alongside CRS-R scores.

For each patient, the measures were normalized for plotting to show the percentage of change relative to the first assessment in that patient. Further, to estimate the stability of each brain-based metric estimated at each assessment, we repeated the above power, connectivity and network analyses 25 times, each time randomly sampling 80% of the retained epochs. The minimum and maximum values obtained over the 25 repetitions were represented as error bars during plotting.

The above data analysis pipeline was implemented using EEGLAB (34), FieldTrip (35), the Brain Connectivity Toolbox (36), and custom MATLAB scripts. The pipeline was automated except for manual checks for and removal of artifactual channels, trials and independent components.

## Results

### Patient 1: UWS to MCS–

Patient 1 (age range: 45–50) was first admitted to hospital nearly 9 years previous to the first hdEEG assessment (3,251 days since injury). Glasgow Coma Scale (GCS) at time of incident was unavailable. At the time of the first assessment, the patient had been in a prolonged UWS with no means of communication, tetraparesis and cognitive difficulties since their injury. There was a noted history of seizures and also some hospital admissions for chest infections. The patient was being treated using Sodium Valproate for management of seizures.

**Figure 2:**
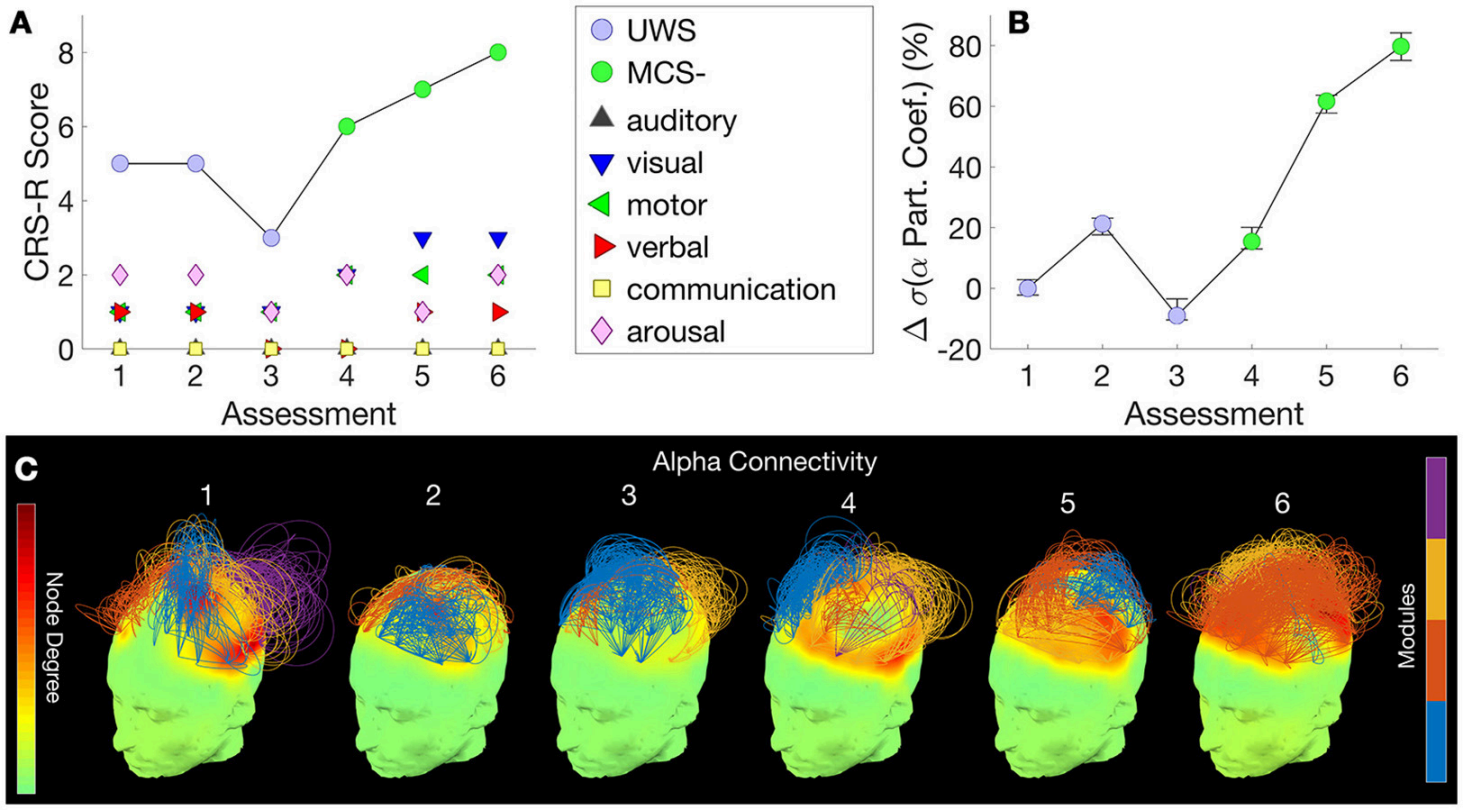
Patient 1 (UWS to MCS-) - CRS-R scores, subscores and diagnosis at each assessment **(A)** are juxtaposed with the normalized standard deviation of participation coefficients estimated from the patient's hdEEG alpha band network at each assessment **(B)**. Consecutive assessments were separated by 3 months. Error bars indicate range of values obtained over 25 repetitions over random subsamples of the original data. **(C)** visualizes alpha band network topographs at each assessment. In each topograph, the color map over the scalp depicts degrees of nodes in the network (left color scale). Arcs connect pairs of nodes, and their normalized heights indicate the strength of connectivity between them. The color of an arc identifies the module to which it belongs, with groups of arcs in the same color highlighting connectivity within a module (right color scale). Topological modules within the network were identified by the Louvain algorithm (16, 18). For visual clarity, of the strongest 30% of connections, only intramodular connections are plotted.

Figure 2A shows this patient's trajectory of CRS-R scores from UWS to MCS–, evidenced by visual pursuit on the visual subscale of the CRS-R. The cross-sectional study in an independent group of patients conducted by Chennu et al. (18) showed that the measure of EEG networks that distinguished UWS from MCS– patients was alpha network centrality, measured as the standard deviation of participation coefficients across the nodes in the network [see Figure 1C in Chennu et al. (18)]. Further, they showed that these hubs were located along a frontoparietal axis of nodes with high connectivity in both locked-in patients and healthy controls [see Figure 1A in Chennu et al. (18)].

Here, within a single patient over a longitudinal period, we observed a visually evident association between their CRS-R score (Figure 2A) and this measure, the normalized alpha band participation coefficient (Figure 2B), as the patient progressed from a UWS to an MCS- diagnosis. Figure 2C plots 3D network topographs visualizing alpha connectivity measured at each assessment. We recorded the highest CRS-R score of 8 at the 6th assessment, when frontoparietal connectivity was most evident in the patient's alpha band network (Figure 2C, far right).

### Patient 2: stable UWS

Patient 2 (age range: 20–25) was admitted to hospital more than 19 months previous to the first hdEEG assessment (584 days since injury). The GCS at time of incident was unavailable. The patient was noted to have widespread intraparenchymal contusions, subarachnoid and subdural hemorrhages as well as base of skull fractures. The patient underwent decompressive craniectomy for raised intercranial pressure and since then, had a cranioplasty. The patient had hydrocephalus and underwent ventriculoperitoneal shunt insertion. The patient's clinical course was complicated with autonomic storming which was managed with Propranolol and Clonodine. At the time of assessment, the patient showed only reflexive behaviors with tetraparesis and no effective means of communication.

**Figure 3:**
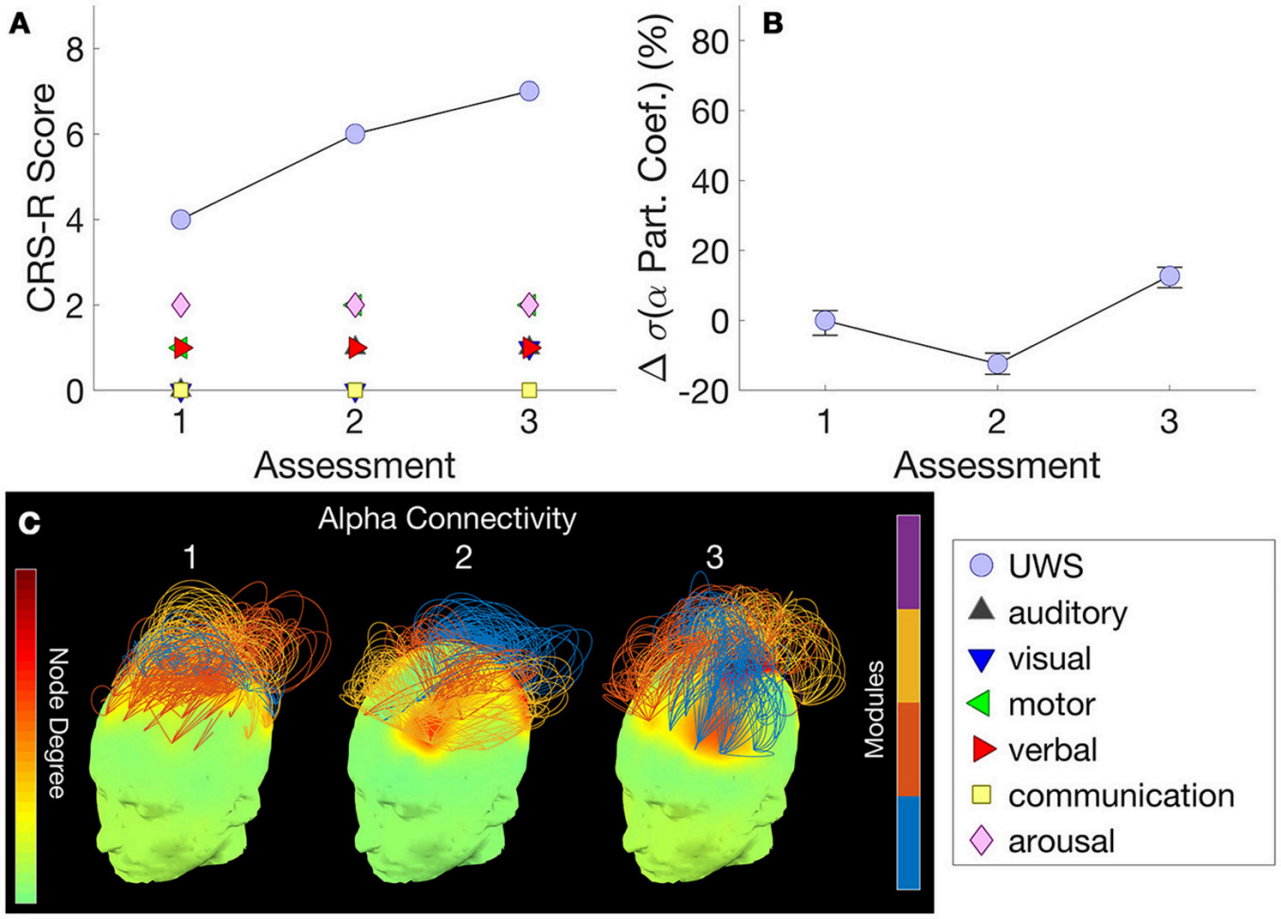
Patient 2 (Stable UWS)– **(A)** shows this patient's trajectory of CRS-R scores and stable diagnosis. Correspondingly, **(B)** demonstrates the relatively consistent standard deviations of the alpha band participation coefficients. **(C)** presents alpha band network topographs at each assessment.

Figure 3A shows this patient's trajectory of CRS-R scores, indicating a diagnosis of UWS throughout. Only three assessments were obtained from this patient, Over these assessments, there was no change observed in CRS-R diagnosis and behavior remained reflexive, with either presence/absence of reflexive responses noted at each assessment in the CRS-R sub-scales. Alongside, there was relatively little variation in alpha network centrality (Figure 3B) across assessments. We observed elevated connectivity during the last assessment (Figure 3C), corresponding with the highest CRS-R score recorded in this patient.

### Patient 3: MCS– to MCS+

Patient 3 (age range: 20–25) was admitted to hospital almost 2 years previous to the first assessment (633 days since injury). This patient was noted to have grade 3 diffuse axonal injury, diffuse subarachnoid hemorrhage and intraventricular hemorrhage as well as bilateral frontotemporal contusions. Their clinical course was complicated by delayed onset rhabdomyolysis, multi-organ failure including acute renal failure for which they had renal replacement therapy. They also suffered a cardiac arrest resulting in hypoxic brain injury.

Approximately 9 months following injury the patient was noted to have a Glasgow Coma Scale (GCS) score of 6/15 and an EEG analysis of event-related potentials completed during their stay in a rehabilitation center showed positive in response to visual stimuli, but auditory ERPs were only positive for stimuli on the right. The patient's clinical course was complicated by seizures and recurrent aspiration pneumonia, supraventricular tachycardia and autonomic storming. They are currently treated with Phenytoin for seizure management.

**Figure 4:**
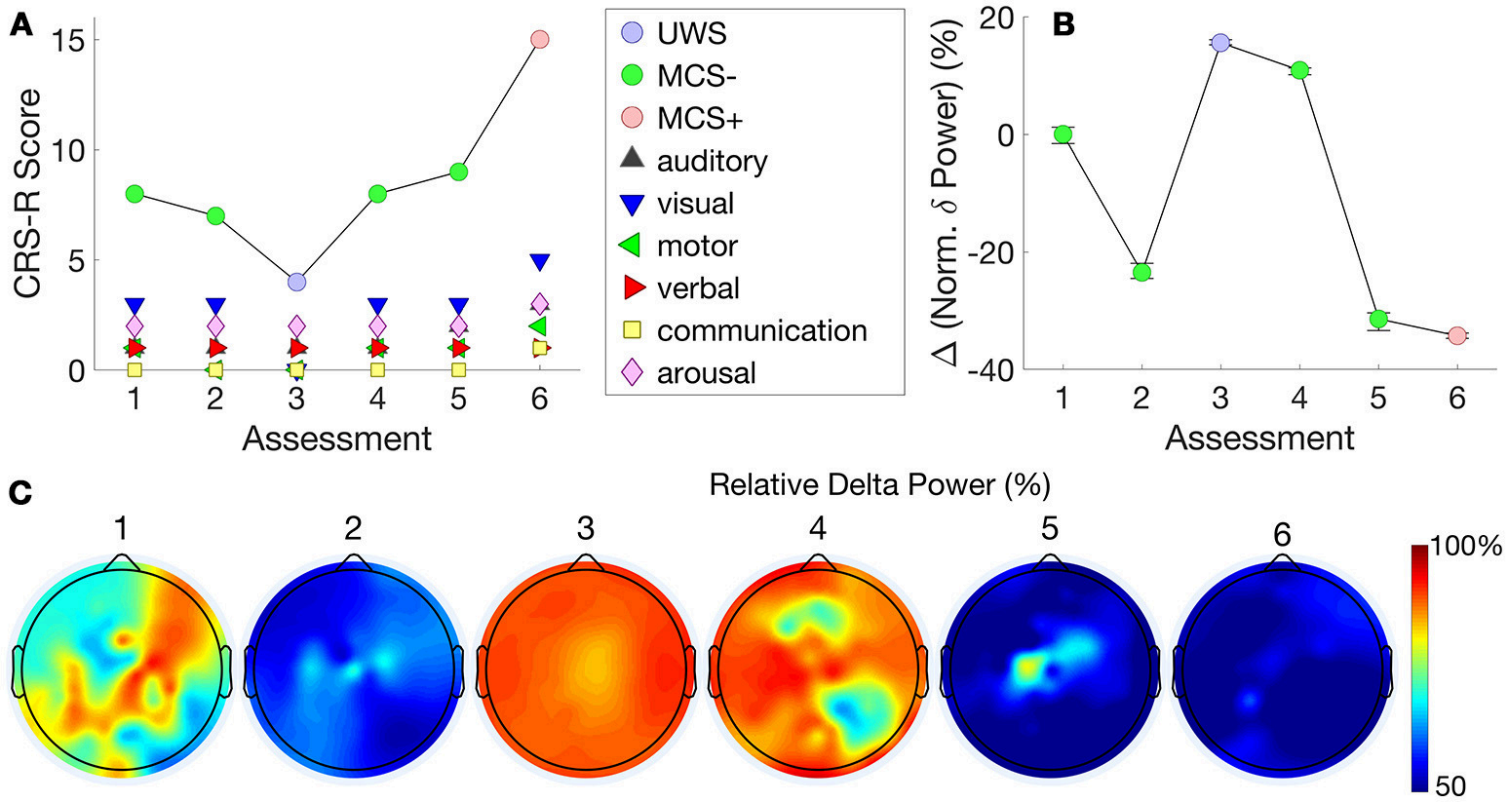
Patient 3 (MCS- to MCS+) - The trajectory of CRS-R scores **(A)** is juxtaposed with normalized delta power, averaged over all channels **(B)**. The relationship between these measures indicates that changes in CRS-R scores were inversely associated with delta power. **(C)** plots normalized delta power topography at each assessment.

Figure 4A shows this patient's trajectory of CRS-R scores from an MCS- to an MCS+ state across time, evidenced by reproducible movement to command on the auditory function scale and inconsistent but intentional attempts to communicate using eye-blinks. Chennu et al. (18) showed that the best hdEEG discriminator of MCS– vs MCS+ patients was delta power [see Figure 1C in Chennu et al. (18)]. In this individual patient, the change in mean normalized delta power validated this finding, inversely mirroring changes in CRS-R over the transition from MCS– to MCS+ (Figure 4B).

Further detail is provided by the delta power topography in Figure 4C. At assessment 3, we recorded a low CRS-R score of 4 and a diagnosis of UWS, as the patient was less responsive (despite the application of deep pressure stimulation recommended by CRS-R guidelines). Consistent with this, delta power was relatively high at almost all channels, and dominated over 90% of total spectral power (Figure 4B). This proportion then dropped to just over 50% at assessments 5 and 6, when we recorded improved CRS-R scores of 8 and 15.

### Patient 4: stable MCS–

Patient 4 (age range 30–35) was admitted to hospital nearly 4 years previous to the first assessment (1,406 days since injury). Their GCS score was 3/15 at the scene. The patient sustained a right acute subdural hemorrhage as well as multiple skull vault fractures. The patient underwent decompressive craniectomy and later developed hydrocephalus that was managed with an external ventricular drain (since removed). The patient's clinical course was complicated with autonomic storming which required treatment with Bisoprolol and Clonodine. The patient also developed sepsis that was treated with intravenous antibiotics. It was noted that further imaging showed a left middle cerebral artery territory infarct.

**Figure 5:**
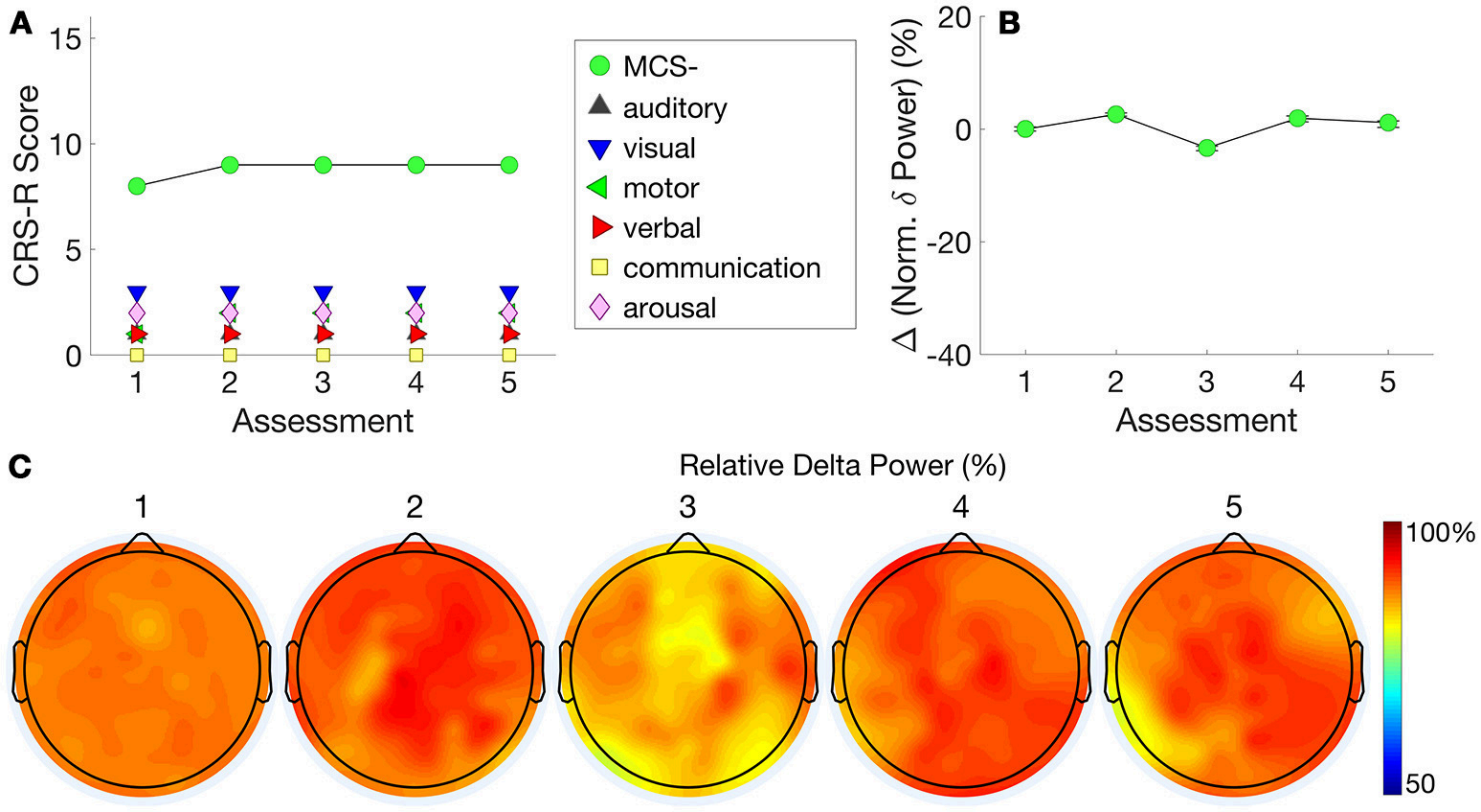
Patient 4 (Stable MCS-)–CRS-R scores **(A)** are presented alongside normalized delta power **(B)**. This patient's stable CRS-R diagnosis is mirrored by stable normalized delta power. **(C)** shows this patient's consistently high delta power topography at each assessment.

Figure 5A shows this patient's very stable trajectory of CRS-R scores. The patient's diagnoses on the CRS-R remained at MCS– throughout the assessments, evidenced by consistent visual pursuit. All other behaviors remained reflexive. In contrast to Patient 3, who showed a progression in normalized delta power alongside a progression in CRS-R scores, Figure 5B shows this patient's stable plateau in delta power that did not change from the first to subsequent assessments. Figure 5C shows that normalized delta power remained high across assessments consistent with a diagnosis of persistent MCS–.

For completeness, Supplementary Figure 1 depicts trajec-tories of alpha network centrality in Patients 3 and 4, and conversely, normalized delta power in Patients 1 and 2.

## Discussion

We have demonstrated a longitudinal approach to the systematic assessment of pDOC patients using a combination of behavioral and brain-based methods at their bedside in a residential neurological center. This is a novel framework that goes beyond most existing research in pDOC, which has typically conducted cross-sectional assessments by transporting patients to specialist hospital centers with advanced neuroimaging facilities. The BETADOC study aims to translate the wealth of neuroscientific evidence generated from these previous studies to the clinical context. This study, to our knowledge, is the first to do so in the UK. Our preliminary findings show that measures of hdEEG networks were correlated with behavioral variations in individual pDOC patients assessed repeatedly at 3-monthly intervals.

### hdEEG assessments in pDOC

We have explored the reliability and stability of hdEEG measures in the contrasting cases considered. Patients 1 and 3 show a progressive transition in CRS-R diagnoses, whilst patients 2 and 4 showed a stable level of behavioral responsiveness over time. Evidence from behaviorally stable patients 2 and 4 lends to the specificity and validity of the hdEEG measures previously identified with cross-sectional analysis (18). Here, the same measures identified as important to detect corresponding within-subject transitions in consciousness were similarly able to demonstrate stability in patients with a persistent and unchanging diagnosis.

In patient 1, whose behavioral scores progressed from UWS to MCS–, we found that the centrality of the patient's alpha band network, as measured by participation coefficients, tracked this improvement longitudinally. This finding was consistent with and extends beyond the cross-sectional analysis in Chennu et al. (18) indicating that standard deviation of alpha participation coefficients was the best discriminator of UWS vs MCS– patients at the group level. The weak alpha connectivity evident in Patient 1's hdEEG network during initial assessments was congruent with a CRS-R diagnosis of UWS. Nevertheless, the patient's hdEEG network evolved over many months of repeated assessments, and we observed the presence of increased frontoparietal connectivity at the 6th assessment, in keeping with an increased CRS-R score and behavioral evidence of consciousness. Broadly speaking, there is considerable evidence linking the presence of robust frontoparietal alpha networks with conscious awareness, from research into other altered states, including sleep (37) and sedation (19, 38) in particular. That hdEEG metrics derived from alpha connectivity can track fine-grained longitudinal changes in behavioral state of an individual pDOC patient, even after a long intervening period since the original brain injury (7 years in case of Patient 1), is valuable new knowledge that speaks to the clinical utility of repeated hdEEG network assessments of consciousness.

### Diverse metrics contribute to discriminative utility

However, this is not to suggest that graph-theoretic metrics are uniquely useful in this context. A relatively simpler estimation of delta band power best discriminated MCS– from MCS+ patients in Chennu et al. (18). Here too, we found that in Patient 3, who progressed from MCS- to MCS+, decrease in delta power was associated with this improvement. In contrast to Patient 3, Patient 4 showed relatively little change in their MCS– state, consistent with a stable level of delta power over multiple assessments. This is in congruence with another independent report of large-scale screening of hdEEG-derived measures by Sitt et al. which showed that different measures were best able to discriminate different states of consciousness (17), and could be beneficially combined. Further, Sitt et al. too reported both positive and negative correlations between hdEEG measures and states of consciousness. More generally, increased power and connectivity in low frequency bands has been reported in pDOC (39), and attributed to partial cortical deafferentation and the consequent intrinsic tendency of such weakly interacting neuronal oscillators to synchronize (40).

### From theory to practice

Taken together, our preliminary findings from the BETADOC project highlight the potential for clinical utility of hdEEG assessments to provide detailed and valuable information about brain activity in individual pDOC patients, across a range of behavioral and clinically relevant stratifications of consciousness. As highlighted earlier, one of the strengths of this project is the longitudinal approach to patient assessment.

Though we have focused on the correspondence between longitudinal changes hdEEG metrics and CRS-R scores here to demonstrate their face validity, the aim of these metrics is not solely to track the CRS-R. Indeed, there are specific data points where there are apparent mismatches between the hdEEG metric and the CRS-R. For example, Patient 3 had similar CRS-R scores at assessments 2 and 4 but different levels of normalized delta power. The magnitude of change in a hdEEG metric, which is unbounded, is not expected to exactly match the magnitude of change in CRS-R, which is by definition bounded between 0 and 23. Rather, we expect that the availability of hdEEG metrics at the bedside could complement the CRS-R by providing brain-based information that cannot be ascertained exclusively via behavioral examination. In doing so, hdEEG assessments could be valuable in reducing the rate of misdiagnosis in practice. As an example, in patients diagnosed with aphasia or with language-related deficits, the behavioral communication necessary for administering the CRS-R might not be possible. In such cases where behavioral assessment is difficult to administer reliably, the entirely passive assessment of conscious state estimated by hdEEG activity could be useful. More generally, having multiple assays of consciousness in individual patients should eventually lead to more accurate estimation, as is known to be the case with repeated CRS-R assessments (41). Indeed, combining diagnostic information from multiple complementary modalities of assessment would promote a rational, consilience-based approach. This is because the ground truth about the patient's subjective conscious state is fundamentally uncertain (42). In the absence of a gold standard for consciousness, convergent information from multiple modalities would increase clinical confidence in the estimation of conscious state. Conversely, divergence, for example where hdEEG indicates a higher conscious state than the CRS-R, can prognosticate the eventual recovery of behavioral consciousness (18).

Another distinct context in which hdEEG assessments add value beyond complementing behavioral assessments is in the identification of patients with the potential for hidden consciousness not expressed in their behavior. This possibility, demonstrated prominently with command following using tennis imagery in seemingly UWS patients (6, 7), has led to the realization that such patients present with a dissociation between cognitive and motor function, rather than unresponsive unconsciousness (43). In these patients, a CRS-R assessment would fail to identify any signs of consciousness due to its absence in behavior. In this context, previous cross-sectional research has highlighted that assessment of hdEEG networks can identify robust alpha connectivity networks in UWS patients who demonstrate evidence of command following with tennis imagery (16). This points to the particular utility of hdEEG assessments in this significant minority of patients who would be misdiagnosed even with systematic behavioral assessment at the bedside.

Finally, the repeatability of hdEEG assessments at the bedside serves as the basis for future work toward demonstrating its utility in the clinical context. Indeed, while the diagnostic and prognostic value of EEG has been highlighted in previous cross-sectional studies of pDOC (8, 16–18, 44, 45), our early evidence speaks to its value within the context of the individual. This is important for advancing beyond the state of the art, from research to clinical practice. Should regular and repeated hdEEG assessments be incorporated into a clinical framework, they could assist with informing clinical decision making on behalf of the patient, addressing an unmet need highlighted in clinical guidelines (1). In particular, as these assessments can be reliably conducted at the bedside, they could be used to identify patients who might benefit from further examination, be it with clinical or neuroimaging methods. Further, we advance the case for exploiting the repeatability of these assessments to evaluate therapeutic and pharmacological interventions. As these often have mixed results in clinical populations (46), hdEEG could be used to better understand the underlying causes of this variable response to treatment. Ultimately, this will contribute to a more evidence-based application of precision medicine tailored to the specific needs and individual histories of pDOC patients.

### Limitations

The findings reported here are preliminary, due to the limited number of patients we were able to include from an ongoing longitudinal study, and hence caution is warranted in their interpretation. In particular, equal numbers of repeated assessments in a large cohort of patients would be ideal for characterizing the trajectory of change in behavior and hdEEG measures. This would enable us to not only arrive at a more accurate behavioral diagnosis, but also identify patients with clear evidence of sustained recovery, in contrast to patients with ongoing fluctuations in behavior. Future research will aim to discover and describe the range of trajectories observed at the cohort level.

Another limitation worth noting is that the patients presented here all had traumatic etiology, hence generalization to other etiologies needs to be the focus of further investigation. Nevertheless, the results presented here are promising as, in this small sample, they suggest that regular and repeated assessment of patients can track variation in CRS-R and brain networks over time. In doing so, our findings point to the potential utility of hdEEG for complementing systematic behavioral assessments at the bedside.

## Author contributions

SC, CAB, JA, DKM, JDP, and PJAH contributed to conception and design of the study; SC developed computational methods and tools. CAB collected the data and organized the database. JA conducted detailed neurological examinations of patients. NR contributed clinical information about patients. CAB and SC conducted the data analysis and wrote the manuscript. All authors contributed to manuscript revision, read and approved the submitted version.

### Conflict of interest statement

The authors declare that the research was conducted in the absence of any commercial or financial relationships that could be construed as a potential conflict of interest.
