# Supplementary material for: Longitudinal Bedside Assessments of Brain Networks in Disorders of Consciousness: Case Reports From the Field
Source: Front Neurol. 2018 Aug 21;9:676. doi: 10.3389/fneur.2018.00676 (PMC6110818; doi:10.3389/fneur.2018.00676)
Supplement: Supplementary file 1 [file Data_Sheet_1.docx]

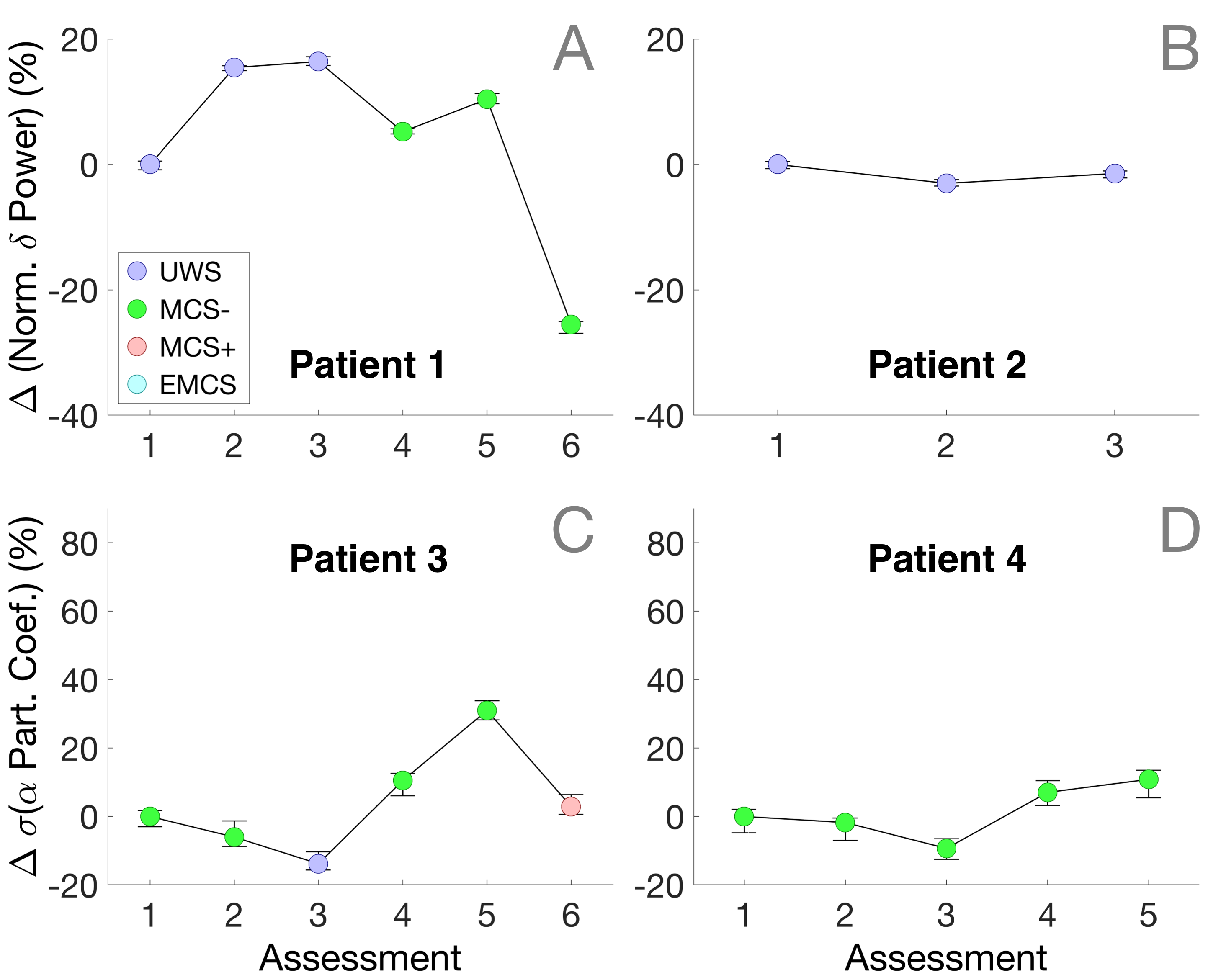


Supplementary Figure 1: Normalised delta band power in Patient 1 (UWS to MCS-; Panel A) and Patient 2 (Stable UWS; Panel B), alongside normalised standard deviation of alpha band participation coefficients in Patient 3 (MCS- to MCS+; Panel C) and Patient 4 (Stable MCS-, Panel D).
